# Epigenome-wide cross-tissue correlation of human bone and blood DNA methylation – can blood be used as a surrogate for bone?

**DOI:** 10.1080/15592294.2020.1788325

**Published:** 2020-07-21

**Authors:** Parvaneh Ebrahimi, Holger Luthman, Fiona E McGuigan, Kristina E Akesson

**Affiliations:** a Department of Clinical Sciences Malmö, Clinical and Molecular Osteoporosis Research Unit, Lund University, Malmö, Sweden; b Department of Clinical Sciences Malmö, Genetics Unit, Lund University, Malmö, Sweden; c Department of Orthopedics, Skåne University Hospital, Malmö, Sweden

**Keywords:** DNA methylation, bone, blood, epigenetics, surrogate tissue

## Abstract

Difficulty in obtaining **bone** tissue is an obstacle to studying epigenetics to understand gene–environment interactions, and their role in disease pathogenesis. Blood is an obvious alternative and in this proof of principle study, our aim was to systematically investigate whether blood is a viable surrogate for bone. We measured epigenome-wide DNA methylation at 850 K CpG sites in matched trabecular bone and peripheral blood collected from the same patients at the same time-point (n = 12 women; 66–85y), to investigate the between-tissue correspondence. What constituted a CpG site with corresponding methylation in both tissues was stringently defined. Only sites highly correlated (r^2^ > 0.74; FDR *q*-value <0.05) and at least 80% similarity in methylation level (Δβ <0.2) between paired samples were retained. In total, 28,549 CpG sites were similarly methylated in bone and blood. Between 33% and 49% of loci associated with bone phenotypes through GWAS were represented among these sites, and major pathways relevant to bone regulation were enriched. The results from this study indicate that blood can mirror the bone methylome and capture sites related to bone regulation. This study shows that in principal, peripheral blood is a feasible surrogate for bone tissue in DNA methylation investigations. As the first step, this will provide a platform for future studies in bone epigenetics, and possibly for larger-scale epidemiological studies.

## Introduction

The traits contributing to age-associated musculoskeletal disorders have a high heritability, estimated from 30% to 80% for bone phenotypes [1,2]. Our comprehension of the underlying genetic architecture of bone traits has advanced thanks in part to genome-wide association studies (GWAS), resulting in the identification of over 100 single nucleotide polymorphisms (SNPs) associated with osteoarthritis, and over 2000 associated with BMD or low impact fracture [3]. Despite these advances, the genetic variants identified cannot fully explain the phenotypic variation, suggesting that environmental factors and factors other than DNA-sequence variation play a role in disease susceptibility [4,5].

Epigenetics refers to variations in phenotype not explained by changes in the DNA sequence [6]. DNA methylation, the addition of a methyl group to the 5ʹ-position of cytosine at so-called CpG sites (where cytosine is positioned before guanine) in the genome, is the most studied epigenetic modification. Alterations in DNA methylation play a major role in the regulation of gene expression. Valuable insights into disease pathogenesis can be acquired by understanding how the methylome is modified [7,8], and by describing disease-related methylation patterns globally or at specific CpG sites [9].

Epigenetic research in the field of bone and musculoskeletal disease is limited [10–13]. Many epigenetic modifications are tissue-specific, and hence it is most informative to use bone itself when searching for the epigenetic signature of a bone-associated trait. However, accessibility of bone tissue and difficulties obtaining appropriate control material present considerable obstacles. This necessitates using alternatives, with peripheral blood the obvious choice as a non-invasive substitute. However, to be able to correctly interpret blood methylation data in relation to bone traits, a clear understanding of the extent to which the bone methylome is reflected in blood, is essential.

A number of studies have investigated DNA methylation in whole blood in relation to bone phenotypes at the epigenome-wide level. While some do not find a consistent association between CpG methylation and BMD [14], others comparing osteoporotic individuals and those with normal BMD have found alternatively none or several CpG sites that differentiated between the groups [15,16]. With these inconsistencies, it is difficult to draw clear conclusions as to what extent bone methylome is reflected in blood.

For other inaccessible tissues, studies have investigated the feasibility of using blood as a surrogate tissue. For instance, studies investigating the concordance between blood and brain methylation levels report 1.4–7.9% of similarity [17,18]. However, to our knowledge, there are no studies on bone tissue which have systematically investigated the feasibility of using blood as a proxy. While we assume there is a correlation between the bone and blood methylomes, it is crucial to substantiate and quantify this. Knowing *what is*, and equally, *what is not* possible is vital for our future understanding. Further down the line, this knowledge will facilitate interpretation of the blood methylome in relation to bone traits. Peripheral blood use will also facilitate leveraging large-scale genetic epidemiological studies, and the eventual identification of DNA methylation biomarkers.

Hence, this is a proof of principle study, to address these gaps in knowledge. Using paired bone and blood samples collected from the same patient at the same time-point, and applying epigenome-wide analysis, we investigated the extent to which the methylomes in bone and blood correspond. The overall aim was to determine if blood is, in fact, a viable surrogate for bone to study DNA methylation in the future.

## Materials and methods

Bone and peripheral blood samples were collected from n = 12 Caucasian women (aged 66–85: mean = 76.3, SD = 6.7) undergoing hip replacement surgery due to osteoarthritis. No exclusions were made based on comorbidity or medications. Bone biopsies consisted of trabecular bone from the exposed proximal femur (inter-trochanteric region) after removal of the femoral head. Biopsies were immediately chopped finely (200–600 mg in total), rinsed with cold saline to remove bone marrow and blood cells before transportation on ice to the lab. Thereafter, biopsies were transferred into Trizol (Life technology^TM^), immediately snap frozen in dry ice/ethanol, and stored at −80°C.

Written informed consent was obtained from the patients, and the study was approved by the Regional Ethics Committee in Lund (LU 957–03).

### Genomic DNA extraction and DNA methylation profiling

Bone samples were thawed, homogenized (Polytron power homogenizer; Thomas Scientific, NJ, USA), and phase separation performed. DNA was isolated using a protocol from Stanford University [19]. From blood, DNA was isolated using QIAamp DNA blood mini kit (Qiagen, Valencia, CA, USA). All DNA was quantified using Nanodrop ND-1000 spectrophotometer (Thermo Scientific™, USA).

In total, the 24 matched bone and blood samples were analysed. For each sample, 500 ng of DNA was bisulphite converted (EZ DNA methylation kit, Zymo Research, CA, USA). DNA methylation profiles were measured using the Human MethylationEPIC BeadChip (Illumina, CA, USA), which covers ~850,000 CpG sites in the genome, including enhancer regions, gene bodies and promoters. BeadChips were imaged using the iScan System (Illumina, CA, USA). Methylation analysis of the samples was performed at SciLife lab, Uppsala, Sweden. Reproducibility of the array is constantly monitored through inclusion of the same QC samples in every sample batch (average reproducibility >0.9).

Samples were randomized to the chips and run in two batches (batch1, n = 14; batch2 n = 10), with matched samples (i.e., bone-blood from the same individual) in the same batch, to avoid intra-individual batch effects (Supplemental Table 1). To quantify the reliability and reproducibility of the methylation measurements and identify any potential bias from batch effect, replicates of one bone sample were run (two replicates on the first chip ‘batch-1ʹ, and one replicate on the second chip ‘batch-2ʹ). Methylation levels were consistent and highly reproducible (Pearson’s *r* > 0.99). The data quality from bone and blood was identical, based on the rate of the probes that were robustly measured and the intensity of the signals.

### Analysis of DNA methylation data

**Figure 1.**
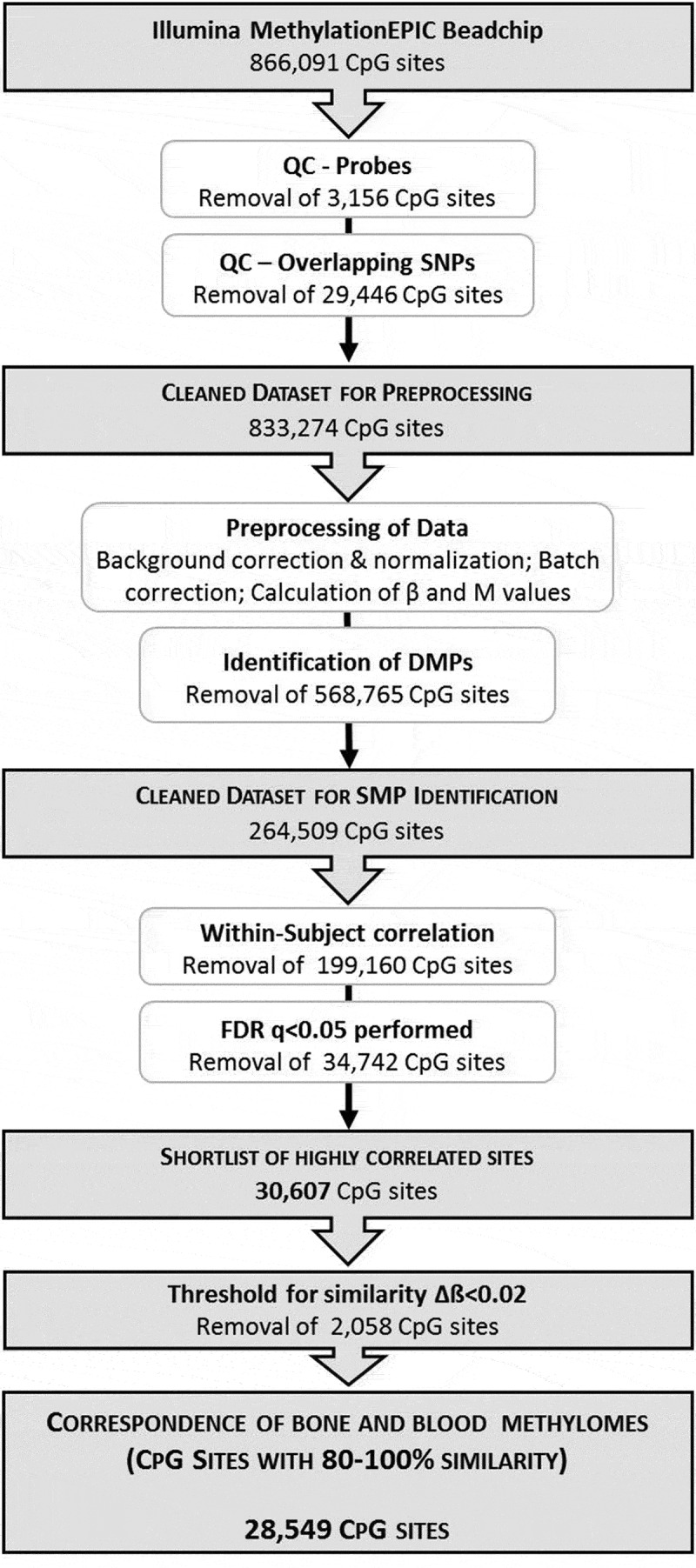
Schematic illustration of the experimental design and bioinformatic analysis strategy to determine the correspondence between bone and blood methylomes

Rigorous approaches were applied to all aspects of the data analysis. A within-subject approach was applied, which takes into consideration the tissue correspondence *at an individual level* rather than the *overall correspondence between tissue* types. Besides, stringent definitions for significance and ‘similarity’ were applied, in order to obtain the most conservative estimate of the between-tissue correlation. The aim was to identify the most concordant sites, minimize the likelihood of SMPs being identified by chance, and narrow the field of capture so far as possible to CpG sites most representative of bone. A flowchart of the data acquisition, preprocessing and analysis strategy is shown in Figure 1.

#### Quality control, preprocessing and normalization

All data preprocessing was performed using R version 3.5.1 [20]. *Minfi* package [21] from Bioconductor [22] was used to import raw intensity data (IDAT) files to R, and for most of the preprocessing steps. *Limma* package [23] was used to calculate multi-dimensional scaling (MDS) plots, based on principal component analysis (PCA) [24].

In the first step, probe-wise quality control was performed to filter out failed probes. A detection *p-*value was calculated for every probe, by comparing the total (*methylated+unmethylated*) DNA signal to the background signal level. Probes with *p*-value>0.01 were excluded (n = 3,156). Sample-specific quality control was also performed, based on the median of the methylated and unmethylated channels. Probes that overlapped with SNPs (n = 29,446) were removed, as these represent direct genetic variations and can affect the downstream analysis. In this cleaned dataset, 833,274 CpG sites remained.

For each CpG site, methylated (*m*), and unmethylated (*u*) intensity values were used to calculate methylation levels as β-values [$\beta =m/\left(m+u+\alpha \right)$] or M-values [$M=\log_{2}\left(m+\alpha /u+\alpha \right)$], where *α* is an offset, customarily 100. β-values represent the percentage of methylation and have a more intuitive biological interpretation, whereas M-values are more statistically valid for data modelling and analysis [25].

For background correction and data normalization, noob (normal-exponential convolution using out-of-band probes) method [26] was performed in *minfi*. To correct for batch effects, ComBat method [27] from surrogate variable analysis (*SVA)* package [28] was used. As all subjects were females, sex chromosomes were not excluded.

#### Identification and removal of differentially methylated CpG sites from the dataset

Sites which were differentially methylated in bone and blood (DMPs) were identified. DMPs were identified by group-mean parametrization, to reduce the effects of genetic variation between patients. A two-step procedure was taken: first, linear models were fit to the M-values using *limma*, including tissue type (variable of interest) and age (adjustment variable) in the design matrix, to minimize confounding from age-associated methylation changes [29]. Thereafter, moderated *t-*statistics were computed for the model. To account for multiple testing, false discovery rate (FDR) analysis was applied by the Benjamini–Hochberg method [30], with adjusted *p-*value (*q-*value) <0.05 considered statistically significant. Second, only sites with an average β-value difference of >20% (Δβ > 0.2) between the paired bone-blood samples were retained. The results of this within-subject differential analysis were compared with a paired-analysis approach, finding identical DMPs by both methods. Proportions of hypo- and hyper-methylated sites in bone relative to blood were calculated, using the average of the M-values difference between matched bone-blood samples.

#### Identification of similarly methylated positions (SMPs) between bone and blood

To identify SMPs, all the statistically differentially methylated CpG sites (*q-*value <0.05) were excluded, in order to restrict the focus to sites potentially concordant between bone and blood (Figure 1). A similarly methylated position was defined as a CpG site with identical (high or low) methylation levels (M-values) in bone and blood of *each individual*. The within-subject correspondence of M-values was calculated for each CpG site using Pearson’s correlation [31] and *t-*test. Following FDR analysis and removal of sites which did not reach statistical significance (adjusted *p-*values (q-value) <0.05), a shortlist of highly correlated sites (0.74<r<0.99) was retained. As a final step, to narrow down these sites to more informative CpGs, a biologically driven criterion for similarity was applied, whereby only sites with maximum 20% difference (Δβ<0.2) between paired samples were retained.

### Annotation of CpG sites and enrichment analysis

Annotation was based on Illumina’s EPIC array annotation data ‘ilm10b4.hg19ʹ. To investigate the enrichment of DMPs and SMPs in gene features, genomic and CpG island coordinates were defined based on the UCSC database (GRCh37/hg19) [32]. TSS200 and TSS1500 are located 0–200 and 200–1500bp upstream of the transcription start site. N- and S-shores are 0–2000bp, respectively, upstream and downstream of CpG islands. N- and S-shelves flank the shores, 2000–4000 bp from CpG islands. These regions are defined in relation to the nearest genes; unmapped sites annotate to open sea. To enable quantitative comparison of the enrichment of CpG sites, the ratio of DMPs and SMPs annotated to each genomic and CpG island coordinates were obtained (i.e., proportional to number of EPIC array probes within each region).

### Proportion of SMPs overlapping known loci for bone phenotypes

The GWAS catalogue was interrogated (22 May 2019) [3], using the keywords: ‘bone density’, ‘fracture’, ‘osteoarthritis’, ‘osteoporosis’ as phenotypes, and associated SNPs and gene names or loci that SNPs were located in or between were extracted. Paediatric traits were not included. For genes with more than one occurrence, only one unique name was retained. Then, a number of the similarly methylated CpG sites that were located within the structure of these selected genes was counted. For comparison, we also interrogated GWAS for a non-bone phenotype (epilepsy).

### Pathway analysis

Pathway enrichment analysis was performed on 28,549 SMPs, using the *missMethyl* R package [33] from Bioconductor [22]. The ‘gene-universe’ was set to the 264,509 CpG sites from which SMPs were identified, and the Molecular Signatures Database (MSigDB) [34] was used as the reference pathway collection.

Pathway enrichment analysis was also performed on a ‘core’ set of highly significantly correlated SMPs (FDR *q*-value<0.005; n = 5,026 CpG sites), to see if it would reveal pathways that would otherwise not be detected.

### Permutation analysis

To confirm that the identified SMPs were beyond what would be expected by chance, permutation analysis was performed using the R package *Rfast* [35], to estimate the proportion of significantly correlated CpG sites under the null hypothesis. Briefly, subject labels of the samples were shuffled (10,000 iterations), effectively breaking the paired nature of the data. At each iteration Pearson’s correlation was calculated, plotting those with FDR<0.05 and Δβ<0.2.

## Results

### Data preprocessing

**Figure 2.**
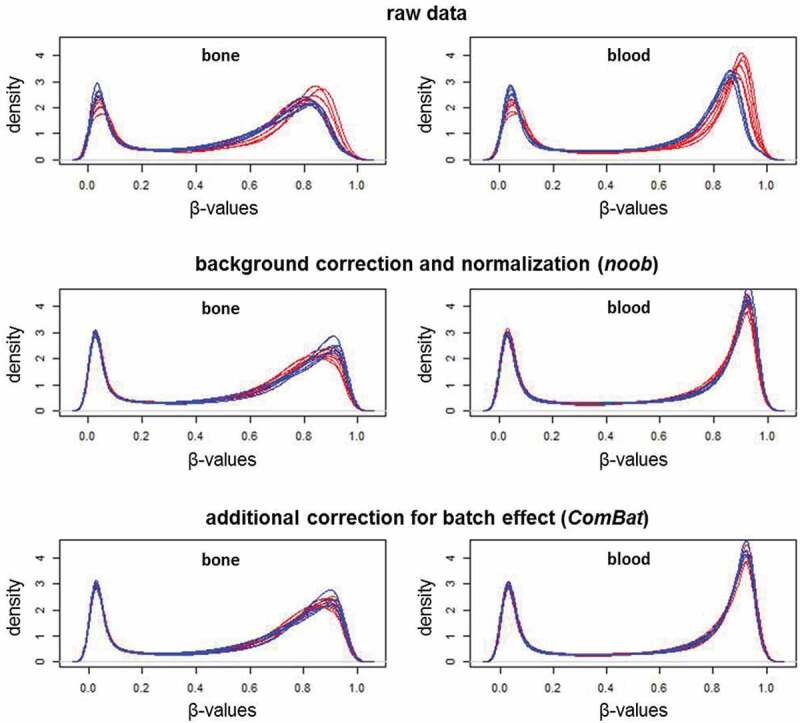
Beta-value density plots of the raw and preprocessed data for bone and blood

**Figure 3.**
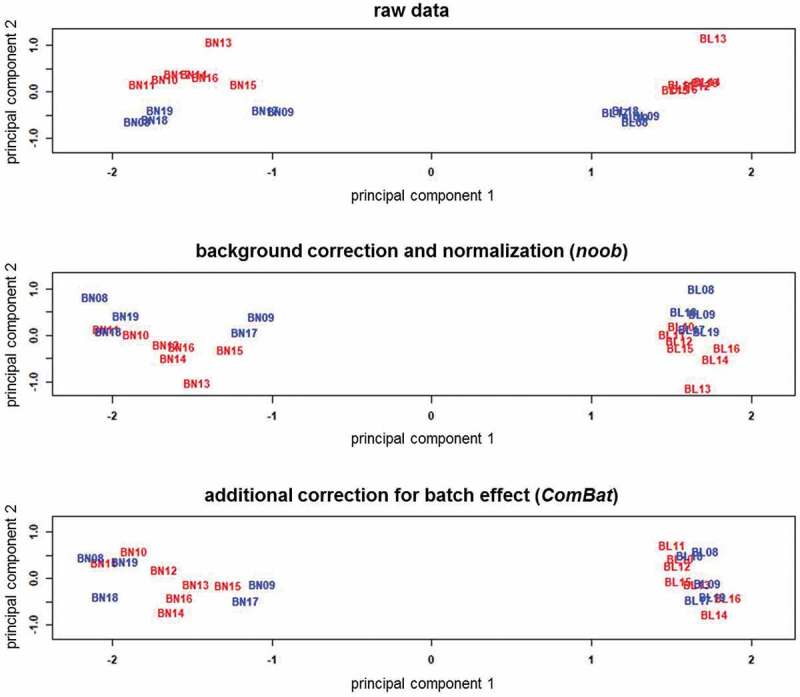
Multi-dimensional scaling plots of the M-values in the raw and preprocessed data for bone and blood

Sample-specific quality control verified the data quality of all samples, and Noob normalization and batch correction further improved data quality, as shown by the β-value density plots (Figure 2). The raw data shows an evident batch effect, whereas the distributions are more consistent in the processed data. As expected, bone and blood have distinct methylation patterns. Bone has a broader profile around the higher methylation levels, while at lower levels, bone and blood profiles look more similar. The MDS plots (Figure 3) show bone and blood are distinctly grouped, even prior to preprocessing, with batch effect reduced after normalization and removed after batch correction.

Plots are coloured by batch (Red, Batch-1; Blue, Batch-2). Batch effect is observed in the raw data, but removed by the preprocessing methods. In the bottom plot, bone and blood show distinct methylation profiles, with bone having a broader distribution around higher methylation levels.

Data points are BN (bone) and BL (blood) with patient ID, coloured by batch (Red-Batch 1; Blue-Batch 2). Tissue type constitutes the main variance in the data; bone and blood samples are distinctly grouped in the raw and preprocessed data. Within the tissues, batch effect is evident in the raw and normalized data based on clustering of the samples, but is removed after batch correction.

### Identification of differentially methylated positions (DMPs)

Prior to SMP identification, in a first step, DMPs were identified. In total, 568,765 sites satisfied statistical significance (*q-*value <0.05). After applying the Δβ>0.2 cut-off, 14,625 sites were considered differentially methylated between bone and blood. The list of the DMPs, as well as the *p-*values, Δβ and annotations are included in Supplemental File 1.

**Figure 4.**
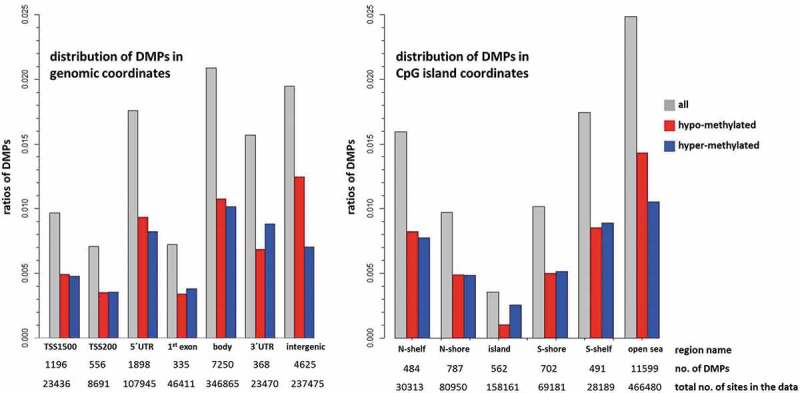
Enrichment of DMPs according to genomic and CpG island coordinates

An overview of the enrichment or depletion of DMPs in genomic regions can indicate which regions are less likely to be informative using blood as a surrogate. DMPs are enriched in gene bodies (2.1% of CpG sites in the array) and intergenic regions (1.9%), 5ʹUTR (1.8%) and 3ʹUTR (1.6%), and under-represented in 1^st^ exon and TSS200 (both 0.7%) (Figure 4). With the exception of the intergenic region, where the proportion of hypo-methylated sites is almost double that of hyper-methylated sites, and the 3ʹUTR where hyper-methylated sites predominate (12.5% higher), the ratio of hypo- and hyper-methylated sites differed only slightly between regions. In relation to CpG island regions, DMPs are enriched in the open sea (2.5%) and shelves (1.6–1.7%) and under-represented in CpG islands (0.4%) and shores (1.0%) (Figure 4). Apart from the open sea region, where the ratio of hypo-methylated sites is higher by 15.2%, and the CpG islands where the ratio of the hyper-methylated sites is higher by 42.3%, the ratios of the hypo- and hyper-methylated sites are almost equal for the shores and the shelves.

The bars show the distribution of differentially methylated CpG sites and the ratios of hypo- and hyper-methylated sites in various regions. The number of DMPs identified and total number of CpG sites in the preprocessed data are also reported. DMPs are depleted in promoter regions and CpG islands. The sum of DMPs in genomic coordinates (n = 16,228) exceeds the total number of DMPs identified (14,625) since some sites have multiple annotations that refer to gene isoforms.

### Identification of similarly methylated positions (SMPs)

After applying a stringent definition of what constituted a similarly methylated site, pair-wise correlation testing of the M-values in the matched bone-blood samples identified a short-list of 30,607 statistically highly correlated CpG sites (*q-*value <0.05). By applying a further final filter and threshold for methylation similarity (Δβ <0.2), a total of 28,549 sites had methylation levels which were at least 80% similar between bone and blood. This equates to 3.4% of the 833,274 CpG sites that passed QC and filtering. The complete list of the SMPs and the correlation coefficients, *p-*values and annotations are included in Supplemental File 2. Among the SMPs, 9,918 (34%) were hypo-methylated (β<0.2) and 2,214 (8%) hyper-methylated (β>0.8).

**Figure 5.**
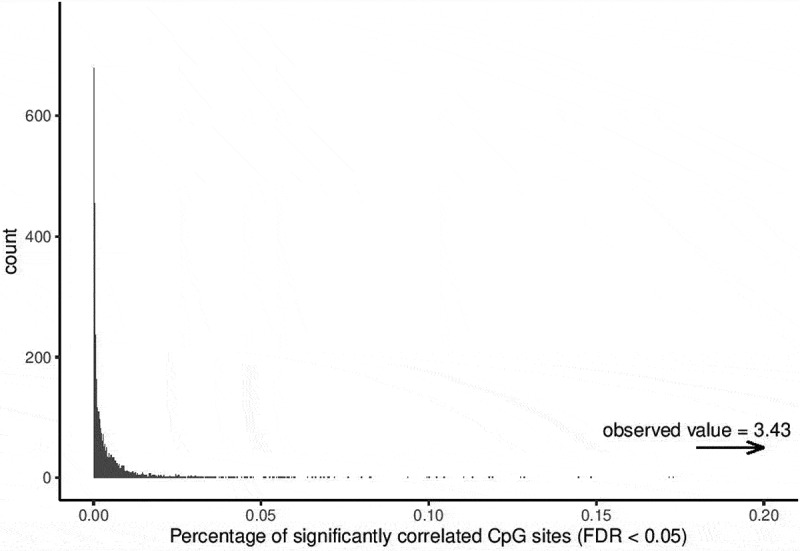
Permutation analysis on the correlation testing to identify similarly methylated positions

Permutation analysis confirmed that the proportion of SMPs is much higher than indicated by permutation, and is much higher than would be expected by chance (Figure 5).

**Figure 6.**
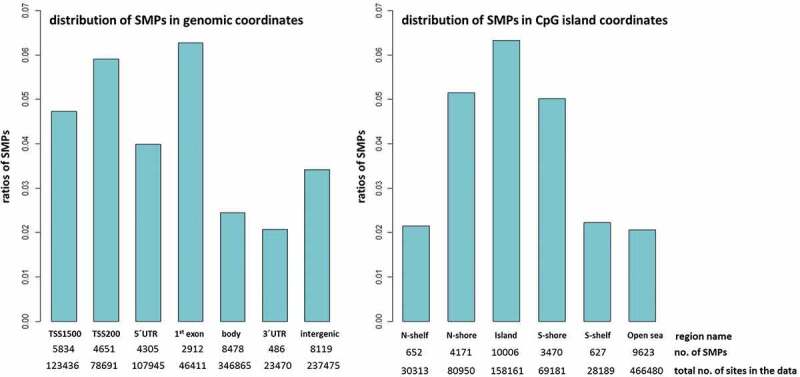
Enrichment of SMP according to genomic and CpG island coordinates

An overview of the enrichment or depletion of CpGs indicates regions for which blood may provide most information as a surrogate. To assess if the SMPs are potentially relevant to gene expression, their genomic coordinates were investigated (Figure 6). SMPs are enriched in the 1^st^ exon (6.3% of CpG sites in the array), TSS200 (5.9%), TSS1500 (4.7%), and 5ʹUTR (4.0%), and under-represented in the 3ʹUTR (2.1%), gene body (2.4%) and intergenic (3.4%) regions. SMPs are enriched in CpG islands (6.3%) and shores (5.0–5.2%) and under-represented in the open sea (2.1%) and shelves (2.2%).

The bars represent ratios of the CpG sites similarly methylated in bone and blood. For each region, the number of the SMPs identified and a total number of CpG sites in the preprocessed data are reported. SMPs are enriched in CpG islands and regions that have regulatory roles in gene expression. The sum of SMPs in genomic coordinates (n = 34,785) exceeds the total number of SMPs identified (28,549) since some sites have multiple annotations that refer to gene isoforms.

### Overlap of SMPs with genetic loci associated with bone phenotype

The next objective was to demonstrate enrichment of bone associated loci among this stringently defined subset of SMPs, and confirm that the blood methylome can capture ‘bone related’ content. The GWAS catalogue contained 558 unique single nucleotide polymorphisms (equating to 411 unique loci) associated with bone phenotypes. Almost 33% of the loci for osteoporosis phenotypes and 49% of known OA loci were represented among the methylation data (Table 1 and Supplemental File 3 for gene lists and complete results). On the other hand, the overlap for epilepsy as a non-bone phenotype was only 18%.

**Table 1.** Number of loci for bone phenotypes extracted from the GWAS catalogue (**A**), number of GWAS loci represented among the similarly methylated positions

	(A) GWAS Catalogue*	(B) Bone-Blood similarly methylated CpG sites	
GWAS Associated Phenotype	N^o^ of GWAS associated genes/loci (SNPs)	N^o^ of overlapping SMPs	N^o^ of GWAS loci represented in the SMPs
Osteoporosis (BMD, Fracture)	310 (472)	242	102 (33%)
Osteoarthritis	101 (86)	129	50 (49%)

*Number of unique SNPs and loci associated with bone phenotypes (excluding paediatric traits) at interrogation (22 May 2019).

**Figure 7.**
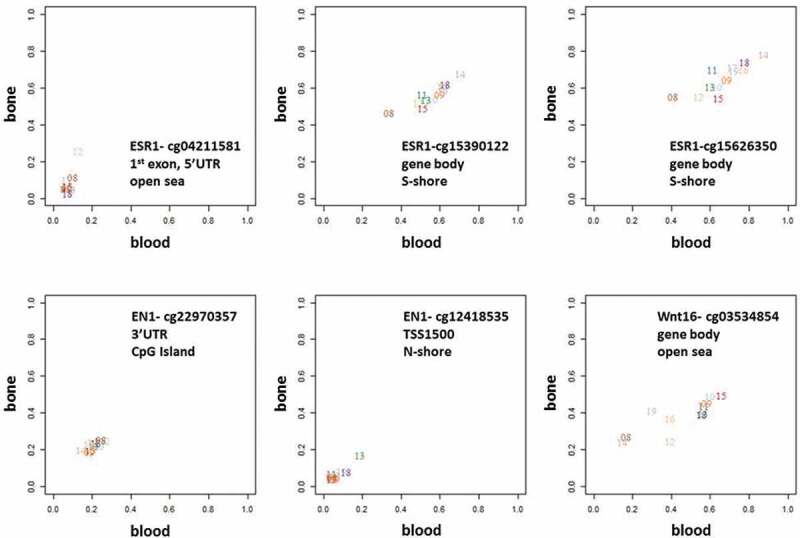
Methylation levels in bone versus blood samples in the SMPs overlapping with 3 examples of genes involved in pathways known to be critical to bone biology – *ESR1, EN1*, and *Wnt16.*

Genes of critical importance for bone biology, inlcuding *ESR1, EN1, Wnt16, DKK1, SMAD3, SOX9, OPG,* and *RANKL*, all contained at least one CpG site (range 1–4) where bone-blood methylation levels were highly correlated. For some sites (Figure 7), there was inter-individual variation in the methylation levels (e.g., cg15390122 in *ESR1*), while for others it was relatively constant between individuals (e.g., cg22970357 in *EN1*).

The legends show the sites names and their genomic and CpG island coordinates. Some of the sites show high inter-individual variation, with methylation levels for bone and blood varying considerably between subjects, whereas other sites have low inter-individual variation and methylation levels are fairly consistent between individuals.

Pathway analysis showed that among the top 20 ranked terms, pathways related to bone regulation were enriched, such as ‘Wnt signalling’ and ‘Oestrogen response’, when including all the SMPs in the analysis (FDR *q*-value<0.05 and Δβ<0.2), shown in Table 2. Some of these key pathways were also enriched among a ‘core’ set of highly significantly enriched SMPs (FDR *q*-value <0.005 and Δβ<0.2), shown in Table 3.

**Table 2.** Pathway enrichment analysis using the molecular signatures database (MSigDB) on all the SMPs (FDR *q*-value of correlation< 0.05, and Δβ<0.2)

MSigDB Term	Number of genes in the term	Number of genes in the SMPs	*p*-value for over-representation
HALLMARK_OESTROGEN_RESPONSE_EARLY	197	141	0,00002
PID_BETA_CATENIN_NUC_PATHWAY	78	57	0,00332
PID_CDC42_REG_PATHWAY	30	25	0,00536
HALLMARK_ANDROGEN_RESPONSE	101	70	0,00673
HALLMARK_UNFOLDED_PROTEIN_RESPONSE	112	73	0,00906
PID_WNT_SIGNALLING_PATHWAY	28	22	0,01016
PID_LKB1_PATHWAY	47	35	0,01251
HALLMARK_UV_RESPONSE_UP	157	101	0,01448
PID_P38_MK2_PATHWAY	21	17	0,02089
PID_BETA_CATENIN_DEG_PATHWAY	18	15	0,02469
NABA_COLLAGENS	43	32	0,02478
PID_HDAC_CLASSII_PATHWAY	33	25	0,02870
HALLMARK_OESTROGEN_RESPONSE_LATE	196	123	0,02882
PID_LPA4_PATHWAY	15	13	0,02917
PID_SYNDECAN_3_PATHWAY	16	13	0,03183
BIOCARTA_CARM_ER_PATHWAY	34	25	0,03857
BIOCARTA_MTA3_PATHWAY	18	14	0,03950
ST_FAS_SIGNALLING_PATHWAY	64	43	0,03950
PID_P53_DOWNSTREAM_PATHWAY	136	86	0,04025
PID_MYC_REPRESS_PATHWAY	62	43	0,04317

**Table 3.** Pathway enrichment analysis using the molecular signatures database (MSigDB) on a ‘core’ set of highly significantly correlated SMPs (FDR *q*-value of correlation< 0.005, and Δβ<0.2)

MSigDB Term	Number of genes in the term	Number of genes in the SMPs	*p*-value for over-representation
NABA_COLLAGENS	43	16	0,00175
PID_P38_MK2_PATHWAY	21	8	0,01268
PID_SMAD2_3PATHWAY	17	7	0,01459
BIOCARTA_VITCB_PATHWAY	11	5	0,02346
HALLMARK_MYOGENESIS	196	41	0,03911
NABA_BASEMENT_MEMBRANES	40	12	0,04971
PID_WNT_SIGNALLING_PATHWAY	28	8	0,05285
NABA_CORE_MATRISOME	263	51	0,06253
BIOCARTA_PTC1_PATHWAY	11	4	0,06925
PID_WNT_NONCANONICAL_PATHWAY	32	9	0,07150
BIOCARTA_ACE2_PATHWAY	12	4	0,07831
PID_AVB3_INTEGRIN_PATHWAY	73	17	0,08961
PID_CIRCADIAN_PATHWAY	16	5,5	0,08970
PID_SYNDECAN_1_PATHWAY	44	11	0,08990
PID_P75_NTR_PATHWAY	67	14,5	0,10464
NABA_PROTEOGLYCANS	31	7	0,10553
BIOCARTA_AMI_PATHWAY	19	5	0,11518
PID_P38_ALPHA_BETA_DOWNSTREAM_PATHWAY	38	9,5	0,11830
ST_GRANULE_CELL_SURVIVAL_PATHWAY	26	7	0,12852
BIOCARTA_INTRINSIC_PATHWAY	21	5	0,13356

## Discussion

Epigenetic markers should contribute to the understanding of disease pathogenesis in bone and in the field of osteoporosis, although difficult to study. While GWAS has provided considerable insight into genes involved in bone regulation, it is through the study of epigenetics that the mechanisms of gene–environment interaction can be elucidated. In view of the difficulties in obtaining viable bone tissue from larger numbers of individuals, it would vastly improve the possibilities to study bone epigenetics if a surrogate tissue could be used. The obvious candidate tissue is peripheral blood.

This study therefore systematically investigated the correspondence between bone and blood methylation profiles in matched samples, as recommended from studies addressing similar issues in brain research [18]. Investigating DNA methylation at more than 850 K sites, 28,549 CpG sites similarly methylated in both bone and blood were identified. A very stringent definition of ‘similarity’ in methylation (80% or more) was applied, in order to focus only on the most highly correlated positions, whether hypo- or hyper-methylated. A within-subject statistical approach and pair-wise correlation testing were applied (since inter-subject approaches using non-matched tissues over-estimate between-tissue correspondence – related to averaging methylation markers across individuals in each tissue type) [18]. In our study, the correspondence between bone and blood equates to 3.4% of all sites in the final dataset. This is in the same range as for other inaccessible tissues such as brain [17,18], although most likely a conservative estimate, based on our approach combining a stringent definition and matched analysis. Among the similarly methylated positions, we had 33–49% overlap with loci robustly associated with bone phenotypes through large-scale GWAS. Compared to a selected non-bone phenotype, epilepsy, for which the overlap was only 18%, bone-phenotypes show a much higher overlap. Many key genes implicated in bone metabolism, including *EN1, ESR1, Wnt16*, and *RANKL* were represented, and major pathways relevant to bone regulation, including Wnt signalling and oestrogen response, were enriched among the similarly methylated positions, which further substantiates the feasibility of our approach.

Reviewing the available bone epigenetic literature, no directly equivalent study exists; *only* bone biopsies or *only* blood in different states of bone health (normal, osteoporosis, osteoarthritis, hip fracture) are used. However, it allowed us to confirm that CpG sites identified in the current study were also identified directly in one of these tissues or as discriminating between bone diseases. In bone biopsies, six of the similarly methylated CpG sites that we identified were also reported to differentiate between hip fracture and osteoarthritis [36], while two sites reportedly differentiated osteoporotic and healthy individuals [12]. These two sites are located in the *TNXB* and *DKK1* genes, and the study also showed that in an independent cohort the *DKK1* site correlated with BMD in blood methylome. From a study using blood alone from osteoporotic and normal post-menopausal women [16], 16 sites were also captured in our study. The fact that we find an overlap between our similarly methylated positions and results from studies comparing bone phenotypes gives credibility to their potential as important regulators in bone metabolism. Importantly, it demonstrates that phenotypic variations in bone are to some extent reflected in the blood methylome.

Even among the similarly methylated positions not directly overlapping known bone-associated loci, hypothetically, some may still lie sufficiently close to impose a distal regulatory role, e.g., situated in enhancers in intergenic regions. Whether they play a role in bone biology will, of course, require further investigation. Overall, given our incomplete understanding of the genetic architecture underlying bone metabolism, the 28,549 similarly methylated CpG sites could include many candidates associated with phenotypic variations, as indeed could many of the DMPs.

The distribution patterns of similarly or differentially methylated positions across the genome provides an outline of tissue similarities and discrepancies. Bone and blood were most similar in regions with regulatory roles in gene expression, i.e., enriched in CpG islands and shores. The density of similarly methylated positions decreased flanking outwards from the islands to shores and shelves; methylation at CpG islands is known to be highly correlated between tissues [37–39]. Similarly, methylated positions were also over-represented in the 1^st^ exon and promoter regions and under-represented in gene body, intergenic and 3ʹUTR; CpG methylation is known to be less variable in the promoter and 1^st^ exon, whereas dynamic in other regions [40–43]. Accordance of our results with the literature demonstrates that our experimental design and data analysis are capturing the ‘right’ biology and inter-tissue epigenetic correspondence.

**Our study has a number of strengths**, the first being the study design, involving elderly females of a limited age range, and matched bone-blood samples collected from the *same patients* at the *same time-points*. Having paired tissue samples is a great advantage when investigating biological correspondence between tissues. When *individual* tissue types derived from *different* individuals are used, inter-subject and within-tissue variability are introduced, and consequently, within-tissue correspondence is overestimated [18,37]. Therefore, by using paired tissue types, we avoid these complications and capture a more accurate picture, without significant confounding from the genetics of the patients. Secondly, we narrowed our focus to include only sites where the methylation levels were highly similar in bone and blood. The cost of this rigour is an underestimation of the reported correspondence, although we consider this beneficial. Thirdly, we performed pathway analysis, whereby even applying an FDR threshold of <0.005, we could demonstrate enrichment of biologically relevant pathways among the SMPs, while a substantial number of GWAS associated loci were also captured. The fact that many of these SMPs were enriched in CpG islands, promoter regions and gene body suggests potential regulatory roles for some of the sites. Fourth, for the statistical analysis, we took different approaches for identifying similarly and differentially methylated sites, all of which are well-established methods, have formerly been used for methylation data, and are reported in the literature. For similarly methylated sites, we used a between-tissue, within-subject approach. This restricted the results only to those sites for which blood reliably predicted methylation in bone on an individual level. On the other hand, for the differential analysis, we took a group-mean approach in order to minimize the effects of inter-individual genetic variations. Interestingly, when we tested the pair-wise approach, the final differentially methylated sites were identical to those picked by the group-mean approach. This could indicate that genetic variation among the subjects has not substantially influenced the results, and that the results are robust. Finally, among the other strengths are the use of the Illumina MethylationEPIC BeadChip, which measures methylation at more than 850 K CpG sites in the genome. Besides higher coverage, an important feature is the inclusion of distal regulatory elements previously lacking [44]. We also extensively tested a number of preprocessing techniques, ultimately choosing those that provided the best data quality, based on methylation density plots and PCA plots.

**Limitations of the study are acknowledged**, the first being the relatively small sample size that was inevitably imposed by the difficulty of obtaining bone tissue – an obstacle for all studies investigating difficult to obtain tissues. However, our sample size is adequately powered to satisfy nominal significance [45]. We have minimized the likelihood of false positives through FDR analysis, restricting our results to larger effect sizes and methylation differences; permutation analysis meanwhile indicates that the reported correspondence is beyond what would be expected by chance. Second, although having biological replicates in the design would have been valuable, due to technical limitations imposed specifically by the difficulty to collect bone samples, it was not possible. On the other hand, technical replicates were incorporated. We also acknowledge as a limitation that validating the SMPs was not possible. Thirdly, we did not specifically correct for cell type heterogeneity, although we acknowledge that both bone and blood are heterogeneous and cell type heterogeneity can affect methylation data. However, there were no significant differences between subjects for blood cell populations (data not shown) [46,47]. Furthermore, our statistical approaches implied that cell type population is not a main source of variation in our samples. Confounding should be further mitigated by the pair-wise correlation analysis of the matched bone-blood samples, which also minimizes confounding from underlying genetic variation. Finally, the lack of healthy controls could be considered a limitation, although this study only investigates correspondence between tissues with no assumptions regarding disease or causality. Hence, we do not consider this a major constraint.

Epigenetics has huge potential to add to our understanding of the mechanisms modulating genetic regulation. The purpose of this study was to test a proof of principal – is blood a feasible surrogate tissue for bone, bone being an inaccessible tissue? In this respect, our systematic analysis of matched bone and blood samples demonstrates that it is feasible. This essential first step will facilitate future understanding of the interplay between the methylome and bone traits. We anticipate our results being useful for a number of purposes. The described similarly methylated sites can be exploited to explore which sites associate with bone phenotypes in population-based studies. In the long term, this could include the possibility of identifying CpG sites as biomarkers for clinically important bone phenotypes. In addition, the investigation of specific genes and the role of selected CpG sites in relation to gene function is possible. The differentially methylated sites may also provide valuable tissue-specific information, and can be probed to identify novel genes and pathways directly or indirectly involved with bone metabolism. In conclusion, the knowledge acquired by our study provides a necessary platform for future studies in bone epigenetics.

## Supplementary Material

Supplemental Material
