# Supplementary material for: Epigenome-wide cross-tissue correlation of human bone and blood DNA methylation – can blood be used as a surrogate for bone?
Source: Epigenetics. 2020 Jul 21;16(1):92–105. doi: 10.1080/15592294.2020.1788325 (PMC7889104; doi:10.1080/15592294.2020.1788325)
Supplement: Supplemental Material [file KEPI_A_1788325_SM5209.zip › Supplemental data legends.docx]

**Supplemental data legends:**

**Supplemental Table 1.** Summary of the information of the patients and the batch design, sorted based on the age (range: 66-85 y) of the patients.

**Supplemental File 2.** The list of the SMPs, summary statistics (correlation values and the adjusted *p-*values of the correlation testing), and the annotation of the SMPs in relation to genomic and CpG island coordinates.

**Supplemental File 1.** The list of DMPs, summary statistics (adjusted *p-*values, and the Δβ for each site), and the annotation of the DMPs in relation to genomic and CpG island coordinates.

**Supplemental File 3.** The gene list for selected bone phenotypes and the overlap of SMPs with these genes.
