# Supplementary material for: Epigenome-wide cross-tissue correlation of human bone and blood DNA methylation – can blood be used as a surrogate for bone?
Source: Epigenetics. 2020 Jul 21;16(1):92–105. doi: 10.1080/15592294.2020.1788325 (PMC7889104; doi:10.1080/15592294.2020.1788325)
Supplement: Supplemental Material [file KEPI_A_1788325_SM5209.zip › Supplemental Table 1.docx]

**Supplemental Table 1.** Summary of the information of the patients and the batch design, sorted based on the age (range: 66-85 y) of the patients.

| **Patient ID** | **Gender** | **Age** | **Batch number** |
| --- | --- | --- | --- |
| 10118 | female | 66 | 2 |
| 10112 | female | 67 | 1 |
| 10109 | female | 68 | 2 |
| 10113 | female | 71 | 1 |
| 10119 | female | 75 | 2 |
| 10115 | female | 77 | 1 |
| 10111 | female | 79 | 1 |
| 10110 | female | 80 | 1 |
| 10117 | female | 81 | 2 |
| 10114 | female | 83 | 1 |
| 10108 | female | 84 | 2 |
| 10116* | female | 85 | 1 |

* Triplicates of **10116 Bone** sample were included; two in the first batch and one in the second batch.
